# Chest Wound Gunshot Management Aided by Cardiopulmonary Bypass: Interdisciplinary Teamwork or “Serendipity”?

**DOI:** 10.3390/reports8040236

**Published:** 2025-11-13

**Authors:** Valentina Tassi, Roland Peraj, Roberto Cirocchi, Valentino Borghetti, Mark Ragusa

**Affiliations:** 1 Thoracic Surgery Unit, Azienda Ospedaliera Santa Maria, 05100 Terni, Italy; v.tassi@aospterni.it (V.T.); rolandperaj92@gmail.com (R.P.); mark.ragusa@unipg.it (M.R.); 2 Department of General and Oncological Surgery, Azienda Ospedaliera Santa Maria, 05100 Terni, Italy; 3 Cardiac Surgery Unit, Azienda Ospedaliera Santa Maria, 05100 Terni, Italy; v.borghetti@aospterni.it

**Keywords:** pulmonary lacerations, penetrating injury of the chest, damage control surgery, cardiopulmonary bypass

## Abstract

**Background and clinical significance.** Penetrating cardiothoracic wounds require prompt treatment in order to decrease mortality and morbidity. Surgical therapy, aimed at bleeding control and removal of damaged tissue, varies widely from the direct suture of parenchymal lacerations to pneumonectomy, which is characterized by high mortality rates. We report our experience with a patient in hemorrhagic shock due to a gunshot wound to the chest, successfully treated by pneumorrhaphy under cardiopulmonary bypass (CPB). **Case presentation.** A 53-year-old man with a gunshot wound to the chest was admitted to our Emergency Department. A bedside ultrasonography revealed left pleural and pericardial effusion. He was hemodynamically instable, so he was immediately transferred to the operating room by the cardiac and Thoracic Surgery teams. Through a median sternotomy approximately 2 L of blood were evacuated and a deep laceration of the left upper lobe was discovered. The massive bleeding could not be controlled, leading to pleural cavity flooding. The surgical team decided to institute emergency CPB and perform lung repair by pneumorrhaphy, under circulatory support. The patient survived and was discharged on p.o. day 20. **Conclusions.** Clinical expertise, adequate instrumental equipment and a high level of interdisciplinary team-work favorably affected the patient’s outcome.

## 1. Introduction and Clinical Significance

Although penetrating cardiothoracic wounds are very common among war casualties, their incidence in non-military scenarios is much less frequent [1]. Nevertheless, these injuries require prompt and specific treatment in order to decrease mortality and morbidity [2]. Surgical therapy of penetrating wounds of the lung, aimed at bleeding control and removal of damaged tissue, widely varies from direct suture of parenchymal lacerations, through parenchymal resections, up to pneumonectomy which is daunted by a mortality rate approaching 100% [3].

We report our experience with a patient in hemorrhagic shock due to a gunshot wound to the chest, successfully treated by pneumorraphy under cardiopulmonary bypass (CPB).

We present the following article in accordance with the CARE reporting checklist.

## 2. Case Presentation

**Figure 1:**
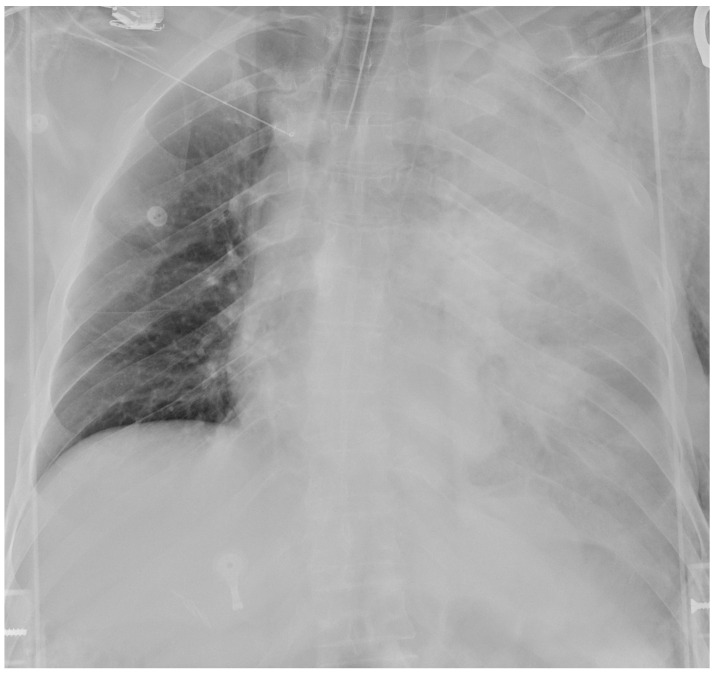
Admission chest film showing massive left hemothorax.

A 53-year-old man with a self-inflicted gunshot wound to the chest was admitted to our Emergency Department. He was hemodynamically unstable, with low values of blood pressure (70/40 mmHg), tachycardia (120 beats per minute) and hypoxemia (peripheral oxygen saturation 80%). Medical examination revealed a 12 mm, round, bullet entry wound at the 3rd intercostal space on the left midclavicular line. An emergency bedside ultrasonography (Point of Care Ultrasound—POCUS) revealed left pleural and pericardial effusion. He was intubated, transfused with one blood bag of iso rhesus, and ev fluid instillation and vasoconstrictor support were started. A chest film showed massive left-sided pleural effusion (Figure 1). Blood samples were as following: hemoglobin concentration 10.6 g/dL, Platelet count 176,000/mmc, Prothrombin Time (PT) 53%, Partial Thromboplastin Time (PTT) 27 s, INR 1.42. His past medical history was characterized by systemic hypertension, treated with Calcium Antagonist 10 mg/die and Acetylcholinesterase inhibitor 10 mg/die. Due to unresponsive hemodynamic instability, the patient was immediately transferred to the operating room by the cardiac and thoracic surgery teams without any further instrumental examination.

During double-lumen reintubation and central vein cannulation, sudden hypotension occurred, requiring emergency surgical incision. A median sternotomy was performed, and the left pleural cavity was accessed, allowing the evacuation of approximately 2 L of blood. The clots were removed, and a deep laceration of the left upper lobe extended to the hilum was discovered; two smaller lacerations were also detected on the anterior surface of the upper and lower lobes of the same lung. The principal vascular branches apparently were not damaged and such observation prompted our attempt to save the lung by avoiding mass procedures at its root. Compression by gauze packing was unsuccessful, let alone any maneuver aimed at isolating the hilar structures. Massive bleeding could not be controlled, leading to pleural cavity flooding. The surgical team decided to institute emergency CPB to attempt lung repair under circulatory support. Direct cannulation of the aorta and right atrium were performed. Heparin (10,000 UI) was administered and CPB started under normothermia. With the heart beating, the blood loss from the injured lung significantly decreased, allowing evacuation of the hemothorax and inspection of the pleural cavity. The described pulmonary lacerations were sutured using continuous 3/0 Polypropylene sutures reinforced with Teflon pledgets. Hemostasis was excellent following these steps. The pleural cavity was thoroughly cleansed and ventilation was restored, allowing gradual CBP discontinuation. Protamine sulfate was administered (CBP time 39’). No active parenchymal bleeding sources were present at this point. Thus, decannulation was carried out. Chest wall repair at the sites of the entry and exit wounds (the latter in the infrascapular area) was performed using continuous Vicryl 1 sutures, after widening and debridement of the skin margins. Hemostasis was checked on the lung and on the thoracic wall, applying hemostatic patches where needed. Two chest drains were inserted in the pleural cavity and a third one in the pericardium. Sternal closure was carried out with steel sutures. Layered suture of the soft tissues.

**Figure 2:**
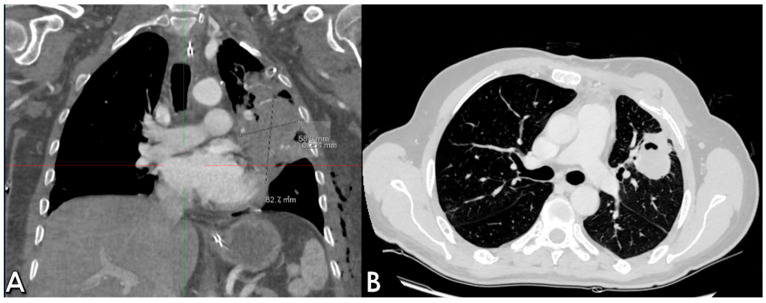
Postoperative sequential chest CT scans showing parenchymal LUL hematoma (**A**) and initial shrinking at CT follow-up (**B**).

**Figure 3:**
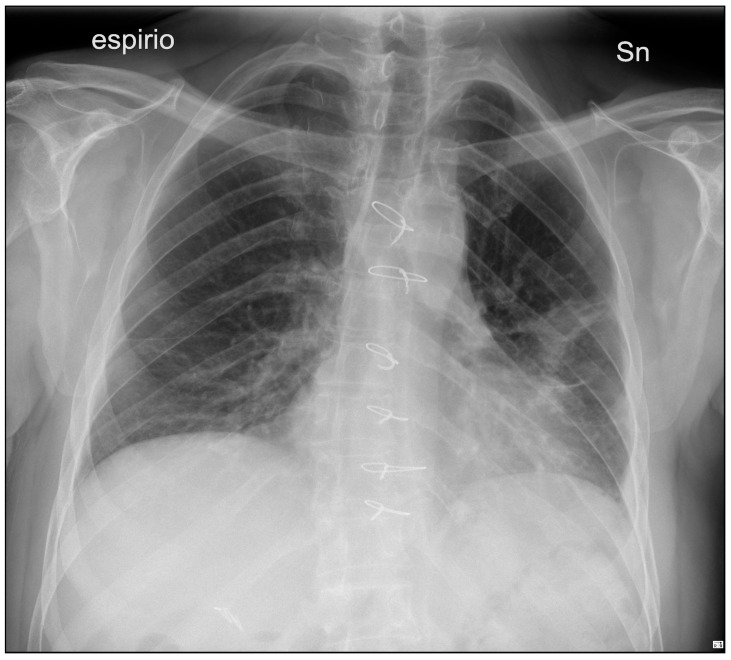
50-day p.o. chest film demonstrating almost total hematoma reabsorption, with satisfactory lung ventilation.

The postoperative course took place in the Intensive Care unit for nine days, during which the patient received blood transfusions (2 units), vasopressor support, antibiotic therapy and steroids. Bronchial toilet was performed twice to free of blood and secretions the tracheobronchial tree. Due to the occurrence of a fever and a rise in the inflammation markers a CT scan was ordered on p.o. day 3, showing an intraparenchymal hematoma (6 cm) in the left upper lobe (LUL), without active bleeding spots (Figure 2A). A conservative approach was chosen and the fever progressively subsided, blood tests gradually normalized and the hematoma progressively shrunk (Figure 2B). The patient returned to the ward on the 9th p.o. day and was discharged from the hospital on p.o. day 20. A chest film obtained a month later revealed almost complete reabsorption of the hematoma, with a very satisfactory lung re-expansion (Figure 3). Data regarding preoperative parameters, intraoperative steps and postoperative events are reported in Table 1.

## 3. Discussion

In civilian scenarios, the incidence of penetrating chest injuries accounts for less than 15% of trauma admissions, but among patients requiring surgical exploration for gunshot wounds, up to 86% of cases present lung lesions [2,4]. Mortality is primarily conditioned by hemodynamic status at admittance and the possibility of performing prompt cardio-pulmonary resuscitation and emergency surgery [2,5]. The management of these patients represents a particular challenge for the emergency team, whose decisions should be driven by hemodynamic (in)stability, extension and location of the wound [2,5].

In our case, the patient was referred to the Emergency Department in hemorrhagic shock, prompting surgical exploration without second level imaging. POCUS had highlighted pericardial effusion (no cardiac tamponade), a finding that we did not confirm intraoperatively. We postulated that massive hemothorax contributed to US overestimation. Due to the site of bullet entry (central chest area between the midclavicular lines) and the presence of pericardial effusion detected by emergency bedside ultrasonography, we decided to perform a median sternotomy to ensure the best exposure to the heart and left anterior pulmonary hilum [2]. A posterolateral thoracotomy would have allowed an accurate exploration of the left pleural cavity and possible clamping of the vascular hilum but could not be performed in such a severely hypotensive patient [5]. Penetrating injuries in the central thoracic area, as described, have the potential for cardiac or great vessel injury, thus are best approached by sternotomy [2].

In penetrating chest trauma, surgery is aimed at the detection of intrathoracic injuries, their repair and consequent hemostasis achievement [1]. Damage control maneuvers such as twisting of the hilum, mass-clamping of the hilar structures or packing of the thoracic cavity, have been proposed to control overwhelming bleeding and postpone major pulmonary resections, but their application is controversial [3]. Most authors advocate for parenchymal sparing techniques, like tractotomy and pneumorrhaphy, as the treatment of choice, leaving lobectomy and pneumonectomy to cases of extended lung destruction due to the high mortality rates related to such procedures in the trauma setting [2,6,7]. In a large nation-based study involving 535 patients, who had a lung resection for “isolated” traumatic injury, the extent of resection turned out to be an independent predictor of mortality (19% for wedge resection, 27% for lobectomy and 53% for pneumonectomy) [8]. Blunt mechanism was associated with prolonged hospital stay, higher rates of complications, disability and mortality due to the combination of respiratory insufficiency, right heart failure and depth of shock [9]. In the present case, after evacuation of two liters of blood and clots, the parenchymal lacerations of the left upper and lower lobes were detected and managed by local compression but bleeding could not be controlled, leading to pleural cavity flooding. As described above, in the case presentation, the principal vascular branches apparently were not damaged and such observation motivated us to try to save the lung. Given that hilar snare through sternotomy resulted ineffective, and considering the very high morbidity/mortality rates of the afore-mentioned emergency maneuvers at the lung root, we decided to establish normothermic CPB which allowed drainage of heart cavities, adequate body perfusion and a significant decrease in bleeding from the pulmonary lacerations. Due to the multiplicity and shape irregularity of lacerations, pulmonary tractotomy was deemed ineffective for hemostasis’ sake, so we preferred performing pneumorrhaphies reinforced with Teflon pledgets.

CPB is safely utilized for complex pulmonary resections in elective settings [10]. Its application in trauma scenarios is established in the case of major thoracic wounds, centrally located, and not otherwise manageable due to the massive hemorrhage. Nevertheless, its implementation is a matter of discussion due to the increased risk of bleeding related to systemic anticoagulation. Alternatively, vv-ECMO (veno-venous extracorporeal membrane oxygenation) has been proposed [6,7]. In our case, the whole procedure was carried out in a critical setting, on a hemodynamically unstable patient. Decisions were guided by the “time factor” and this holds true also for the choice of starting CPB by means of heart-lung machine, immediately available on-site. Indeed, the staring of vv-ECMO surely would have required more complex and time-consuming steps, unfeasible in such a scenario. Finally, only larger hospitals are equipped with extracorporeal circulation systems and feature a dedicated staff trained in central or peripheral cannulation on call 24/7 [6]. Out of large dedicated Trauma Centers and military battlefield health support facilities, surgeons facing penetrating thoracic injuries, indeed, are organ-specialized with a variable training level in operative trauma management [4].

In the present case, we balanced the pros and cons of applying CPB. Since the patient had no coagulopathy and the chest was the only assessed on-site injury, the risk of worsening hemorrhage in other sites appeared to be limited. As expected, extracorporeal circulatory support diminished the bleeding from pulmonary lacerations, allowing us to perform an accurate lung parenchymal repair without any resection.

## 4. Conclusions

In our opinion this case highlights the pivotal role of an interdisciplinary approach to such complex patients, specifically in the emergency setting when decisions are taken on a “moment by moment” basis. The role of a multidisciplinary team was embodied by the ability to make fast and effective decisions requiring different expertise in a coordinated and mutually cooperative atmosphere. Even in non-military hospitals, dedicated training programs aimed at improving cardiothoracic and vascular skills in the management of trauma should be available. In conclusion, clinical expertise, adequate instrumental equipment and a high level of interdisciplinary communication among surgical and anesthesiologic teams, rather than “serendipity”, seem to have affected the favorable outcome of the patient we described.

## Figures and Tables

**Table 1:** Preoperative parameters, intraoperative steps and postoperative course of the case reported.

**Emergency Department**	**Parameters**	Blood pressure: 70/40 mmHg Heart rate: 120 beats per minute Peripheral oxygen saturation: 80%	**Management** Endotracheal intubation Transfusion with one blood bag Intravenous fluid instillation Vasoconstrictor support
	**Medical examination**	12 mm, round, bullet entry wound at the 3rd intercostal space on the left midclavicular line	
	**POCUS**	Left pleural and pericardial effusion	
	**Chest X-rays**	Massive left-sided pleural effusion	
	**Blood samples**	Hemoglobin concentration: 10.6 g/dL Platelet count: 176,000/mmc Prothrombin Time: 53% Partial Thromboplastin time 27 s INR 1.42.	
**Operating room**	**Steps:** - Double-lumen reintubation and central vein cannulation - Median sternotomy - Evacuation of 2 L of blood and detection of pulmonary lacerations - Compression by gauze packing (unsuccessful) - Maneuvers aimed at isolating the hilar structures (unsuccessful) - Direct cannulation of the aorta and right atrium, Heparin administration and start of CPB under normothermia - Suture of pulmonary lacerations using continuous 3/0 Polypropylene sutures reinforced with Teflon pledgets - Restoration of ventilation, CBP discontinuation, administration of Protamine sulfate and decannulation - Chest wall repair at the sites of the entry and exit wounds using continuous Vicryl 1 sutures, drains insertion and sternal closure		
**ICU**	**Day 1–2**	- Blood transfusions (2 units) - Bronchial toilet - Vasopressor support, antibiotic therapy and steroids	
	**Day 3–8**	- Occurrence of fever and rise in the inflammation markers - Chest CT scan: intraparenchymal hematoma in the LUL without active bleeding - Conservative management	
**Ward**	**Day 9–20**	- Defervescence - Blood tests normalization - Hematoma shrinking	

## Data Availability

The original contributions presented in this study are included in the article. Further inquiries can be directed to the corresponding author.
